# Machine learning assisted rational design of antimicrobial peptides based on human endogenous proteins and their applications for cosmetic preservative system optimization

**DOI:** 10.1038/s41598-023-50832-8

**Published:** 2024-01-10

**Authors:** Lizhi Yue, Liya Song, Siyu Zhu, Xiaolei Fu, Xuhui Li, Congfen He, Junxiang Li

**Affiliations:** 1 Key Laboratory of Cosmetic of China National Light Industry, School of Light Industry Science and Engineering, Beijing Technology and Business University, Beijing, China; 2 School of Chemistry and Chemical Engineering, Qilu Normal University, Shandong, China; 3 AGECODE R&D Center, Yangtze Delta Region Institute of Tsinghua University, Zhejiang, China; 4 Harvest Biotech (Zhejiang) Co., Ltd., Zhejiang, China; 5 Zhejiang Provincial Key Laboratory of Applied Enzymology, Yangtze Delta Region Institute of Tsinghua University, Zhejiang, China

**Keywords:** Biotechnology, Computational biology and bioinformatics, Microbiology

## Abstract

Preservatives are essential components in cosmetic products, but their safety issues have attracted widespread attention. There is an urgent need for safe and effective alternatives. Antimicrobial peptides (AMPs) are part of the innate immune system and have potent antimicrobial properties. Using machine learning-assisted rational design, we obtained a novel antibacterial peptide, IK-16-1, with significant antibacterial activity and maintaining safety based on β-defensins. IK-16-1 has broad-spectrum antimicrobial properties against *Escherichia coli*, *Staphylococcus aureus*, *Pseudomonas aeruginosa*, and *Candida albicans*, and has no haemolytic activity. The use of IK-16-1 holds promise in the cosmetics industry, since it can serve as a preservative synergist to reduce the amount of other preservatives in cosmetics. This study verified the feasibility of combining computational design with artificial intelligence prediction to design AMPs, achieving rapid screening and reducing development costs.

## Introduction

The cosmetics industry is one of the most rapidly developing sectors worldwide^1^. Preservatives are essential ingredients in cosmetic products that inhibit microbial growth and prevent spoilage^2^. Common preservatives include parabens, formaldehyde releasers, isothiazolinones, phenoxyethanol, and organic acids^3^. However, preservatives are among the most common allergens in cosmetics^4^. Some preservatives can cause cosmetic contact dermatitis^5^, such as methylisothiazolinone (MI)^6^, methylchloroisothiazolinone/methylisothiazolinone (MCI/MI)^7^, parabens^8^, and formaldehyde releasers^9^. Furthermore, certain preservatives in leave-on cosmetics may disrupt skin microbiota balance^10^. Cosmetic products with low or no preservatives have gained popularity. While combinations of various preservatives represents a current method of improvement^11^, it cannot fundamentally solve this problem. Therefore, there is an urgent need for safe, potent, broad-spectrum antimicrobial agents.

Other substances present in cosmetic products that also have antibacterial effects are known as ‘preservative synergists’^8^. Mixing preservatives and preservative synergists effectively decreases the concentration of allergenic preservatives. Certain antibacterial substances, such as glycolipids^12^ and new antimicrobial nanomaterials^13–15^, may be used as preservative synergists. However, there are few reports on the development of biomimetic antimicrobial peptide derived from human endogenous proteins for use as cosmetic preservatives.

Antimicrobial peptides (AMPs) are oligopeptides with varying numbers of amino acids (from 5 units to > 100 units)^16^. AMPs are important components of the innate immune system in many organisms and provide the first line of defense against various pathogens^17^. AMPs exhibit various activities against Gram-negative and Gram-positive bacteria, fungi, mycobacteria, and some enveloped viruses^18,19^. Most AMPs can be classified into four groups according to their secondary structure: α-helical, β-sheet, loop, and extended peptides^16^. Among these structural groups, α-helix and β-sheet structures are more common^20^. α-helical peptides are the most studied AMPs to date. In α-helix structures, the distance between two adjacent amino acids is around 0.15 nm, and the angle between them with respect to the centre is around 100° from the top view^21^. Human β defensin type 3 (hBD-3) is a cysteine-rich small antibacterial peptide with broad-spectrum antimicrobial activity and may play an important role in innate epithelial defense. Because hBD-3 kills bacteria by disrupting the cell membrane, which is relatively stable in chemical composition, its antibacterial activity will not cause drug resistance^22^. Thus, hBD-3 could serve as a potential AMP for designing novel preservatives. However, natural AMPs are large protein molecules that are unstable in various in vitro environments, which limits their applications. Therefore, developing biomimetic AMPs with improved efficacy, greater stability, and lower costs is challenging. Machine learning (ML) is described as the capacity of a system to learn from problem-specific training data to automate the process of analytical model building and solving associated tasks^23^. ML greatly improves work efficiency and accuracy. Driven by increasing computer power and algorithmic advances, ML has become a powerful tool for finding data patterns^24^, and it is widely applied in various fields, such as biology^25,26^, medicine^27,28^, healthcare^29^, environment^30^, fuel cells^31^ and energy^32^. ML methods can also be used to develop new AMPs. As more insights are gained into the antimicrobial mechanism, sequence, and structure, ML can gather information to expedite AMP screening and design^33–35^.

The primary objective of this study was to develop safe and potent broad-spectrum antimicrobial agents to reduce the use of preservatives in cosmetics. In this study, the artificial antibacterial peptide was designed based on hBDs through computational simulation and artificial intelligence (AI) based ML tools, and their antibacterial and haemolytic properties were predicted.

## Methods

### Designing antimicrobial peptides

AI deep learning based on prediction algorithms and rational molecular design has been used to develop new biomimetic AMPs based on endogenous human proteins^36–38^. Specifically, short analogues of β-defensin were used as template peptides and helical wheel projection was conducted. Negatively charged amino acid residues, including Asp (D) and Glu (E), were substituted with positively charged amino acid residues, including Lys (K) and Arg (R), to increase the net positive charge. Several residues were adjusted such that the hydrophobic and hydrophilic residues were located on opposite sides of the helical surface to improve the pore-forming ability of the microbial membrane^33^. The *ab-initio* three-dimensional structures of peptides were studied to support the α-helical conformations. Peptides were predicted for their antimicrobial ability by some AI programs^39–42^. For safety reasons, the haemolytic activity was also predicted using an available AI algorithm^43^. Antimicrobial activity was predicted using the Antimicrobial Peptide Scanner version 2 (https://www.dveltri.com/ascan/v2/ascan.html), and haemolytic activity was predicted using HAPPENN (http://research.timmons.eu/happenn). All the new top-scoring biomimetic peptides were synthesised and screened for their microbial inhibitory activity.

### Disk diffusion test

Four types of bacteria and fungi, namely, *Staphylococcus aureus*, *Escherichia coli*, *Pseudomonas aeruginosa*, and *Candida albicans* (ATCC, Manassas, VA, USA)*,* were cultured under standard conditions. *Candida albicans* was cultured with Sabouraud glucose broth medium (Solarbio Life Science, Beijing, China) at 30 °C and 200 rpm; *Staphylococcus aureus*, *Escherichia coli*, and *Pseudomonas aeruginosa* were cultured with Luria Broth (LB) liquid medium (Solarbio Life Science, Beijing, China) at 37 °C and 200 rpm. The paper disks containing 30 μg Kanamycin (Beyotime Biotechnology, Shanghai, China) and 30 μg Amphotericin (Beyotime Biotechnology) were assigned as the positive control, and the medium was assigned as the negative control. The paper disks incorporated with 30 μg of AMP were deposited on the surface of the agar plates pre-inoculated with the bacterial strain to be tested. Specifically, 60 μg AMPs were used to test against *Candida albicans*. The agar plates were incubated for 24 h under appropriate conditions. *Candida albicans* was cultured in Sabouraud glucose agar medium in an inverted incubator at 30 °C for 12 h, and the remainder was cultured in LB solid medium in an inverted incubator at 37 °C for 12 h. The diameter of the inhibition zone was measured for statistical analysis. Each test was performed in triplicates.

### Minimum inhibitory concentration

Four types of bacteria and fungi–*Staphylococcus aureus*, *Escherichia coli*, *Pseudomonas aeruginosa*, and *Candida albicans*–were cultured under standard conditions. *Candida albicans* was cultured in Sabouraud glucose broth medium (Solarbio Life Science, Beijing, China) in a static incubator at 30 °C for 12 h, and the remainder were cultured with LB liquid medium (Solarbio Life Science, Beijing, China) in a static incubator at 37 °C for 12 h. The bacterial order of magnitude was 10^4^/mL–10^5^/mL. A series of two-fold dilutions were performed from the stock solution to obtain different concentrations of peptides from 250 to 1.95 μg/mL, with a total volume of 200 μL per well in a 96-well plate, and then assayed for absorbance at 540 nm. This medium was used as the solvent and was designated as the negative control. Approximately 1 × 10^4^ cells were incubated in a medium containing peptides. After incubating the cells at the appropriate temperature for 24 h, the minimum inhibitory concentration was the lowest peptide concentration that limited visible microbial growth.

### Hemolytic activity

For the haemolysis studies, rabbit erythrocytes (Fenghui Biology, Hunan, China) were obtained from healthy blood donors. Blood samples mixed with 0.25% potassium oxalate were immediately separated by centrifugation at 1500 rpm for 10 min. Subsequently, the cells were washed three times with phosphate buffered saline (PBS, Yuanpei Biology, Shanghai, China) and diluted to a 5% suspension with PBS. 250 μL of erythrocyte stock dispersion was mixed with 250 μL of PBS containing different concentrations of AMPs and incubated at 37 °C for 1 h. Intact erythrocytes were removed by centrifugation at 10,000 rpm for 5 min. The absorbance of the resulting supernatant was measured at 540 nm. PBS was used as a negative control (0% lysis), and 0.1% SDS (Solarbio Life Sciences) was used as a positive control (100% lysis). The haemolysis rate was determined using Eq. (1).

1 $$Haemolysis\; Rate\; (\% ) = \frac{{D_{test} - D_{nc} }}{{D_{pc} - D_{nc} }} \times 100\%$$

where *D*_*test*_, *D*_*nc*_, and *D*_*pc*_ are the 540 nm absorbance of the tested sample, negative control, and positive control, respectively.

### Cytotoxicity

HaCaT cells (Fenghui Biology, Hunan, China), a long-lived, spontaneously immortalised human keratinocyte line, was grown in Dulbecco's Modified Eagle Medium (Wisent Bioproducts, Quebec, Canada) supplemented with 10% heat-inactivated foetal bovine serum (FBS, Invitrogen, Carlsbad, CA, USA) and 1% penicillin–streptomycin (Langke Biology, Suzhou, China) at 37 ℃ and 5% CO_2_ until 80–90% of fusion was measured. The experiments were performed in 96-well plates with a final volume of 100 μL of medium per well. HaCaT cell proliferation was tested using the cell counting kit-8 assay (Dojindo Laboratories, Tokyo, Japan). The cells were seeded at a density of 0.5 × 10^5^–1.0 × 10^5^ cells/mL and grown at 37 ℃ until 80–90% fusion was measured. Subsequently, samples at specific concentrations were added to the medium. After incubating the cells for 24 h, all media were removed, and the cells were washed three times with PBS. Next, the cells were incubated for 4 h with fresh medium (100 µL/well) containing 10 µL of the probe. The absorbance was read at 450 nm using a microplate reader.

### Antimicrobial assay in products

The basic preservation system contained 1.8% 1,3-propanediol, 0.09% 1,2-Hexanediol, and 0.036% capryl hydroxamic acid. In the synergy test, the 5 μg/mL antimicrobial peptide was mixed with the preservative system and compared with the preservative system without antimicrobial peptide. Four types of bacteria and fungi–*Staphylococcus aureus*, *Escherichia coli*, *Pseudomonas aeruginosa*, and *Candida albicans*–were cultured under standard conditions. *Candida albicans* was cultured with Sabouraud glucose broth medium (Solarbio Life Science, Beijing, China) at 30 °C and 200 rpm; *Staphylococcus aureus*, *Escherichia coli*, and *Pseudomonas aeruginosa* were cultured with LB liquid medium (Solarbio Life Science, Beijing, China) at 37 °C and 200 rpm. Following culturing, 1.0 × 10^6^ CFU/mL was added to the cosmetic products and mixed thoroughly. At hour 2 (day 0), day 3, day 7, day 14, and day 22, 1.0 mL of the product was collected and neutralised for 30 min using a 9 mL neutraliser. The 1.0 mL of the neutralised product was centrifuged at 12,000 rpm for 5 min to remove the suspension. The product was resuspended in 0.2 mL of fresh medium, and the CFUs were calculated using the spreading plate method. CFU refers to the number of individual colonies of any microorganism that can grow on a plate of media. The standard unit of measure for CFU is the number of culturable microorganisms present per 1 mL of culture (CFU/mL). The colony forming units per mL (CFU/mL) was determined using Eq. (2).

2 $${\text{Colony}}\;{\text{Forming}}\;{\text{Units}}\;{\text{per}}\;{\text{mL}}\;{\text{(CFU}}/{\text{mL)}} = \frac{N}{d} \times V$$

where N is the number of colonies on the counting plate; d is the sampling volume (mL); V is the dilution multiple.

## Results

### The design of antimicrobial peptides

**Figure 1:**
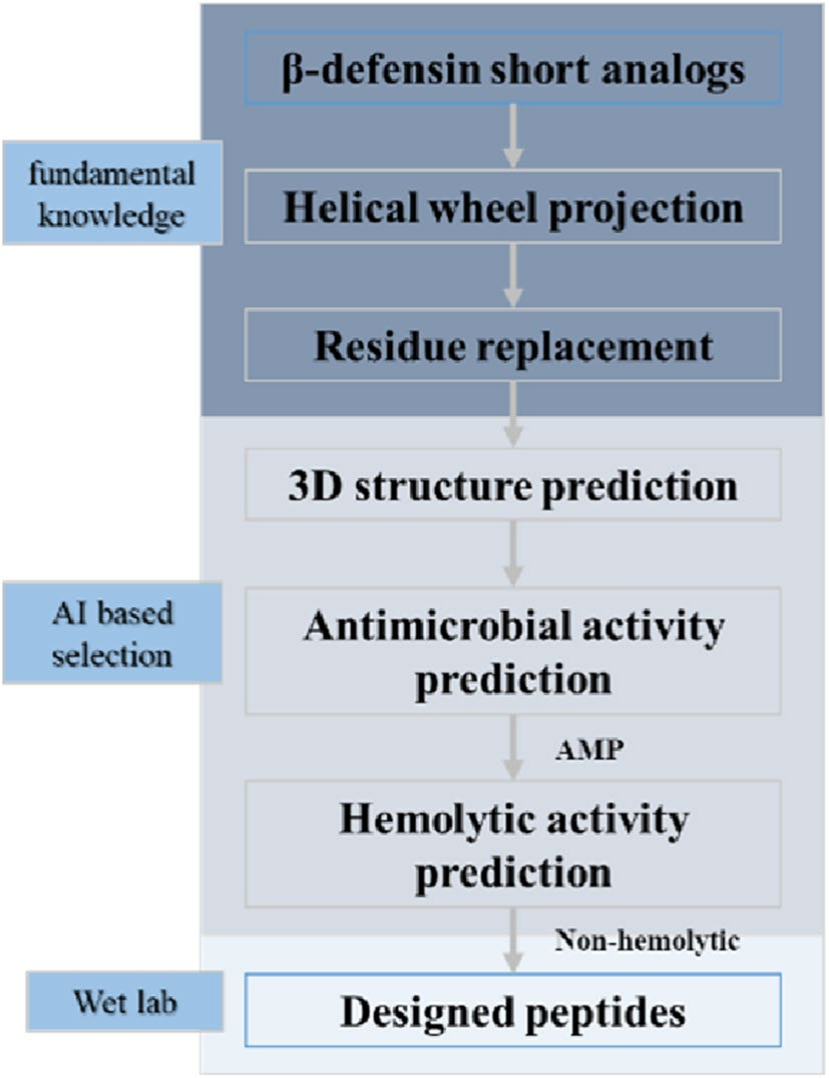
Workflow of antimicrobial peptide design.

**Figure 2:**
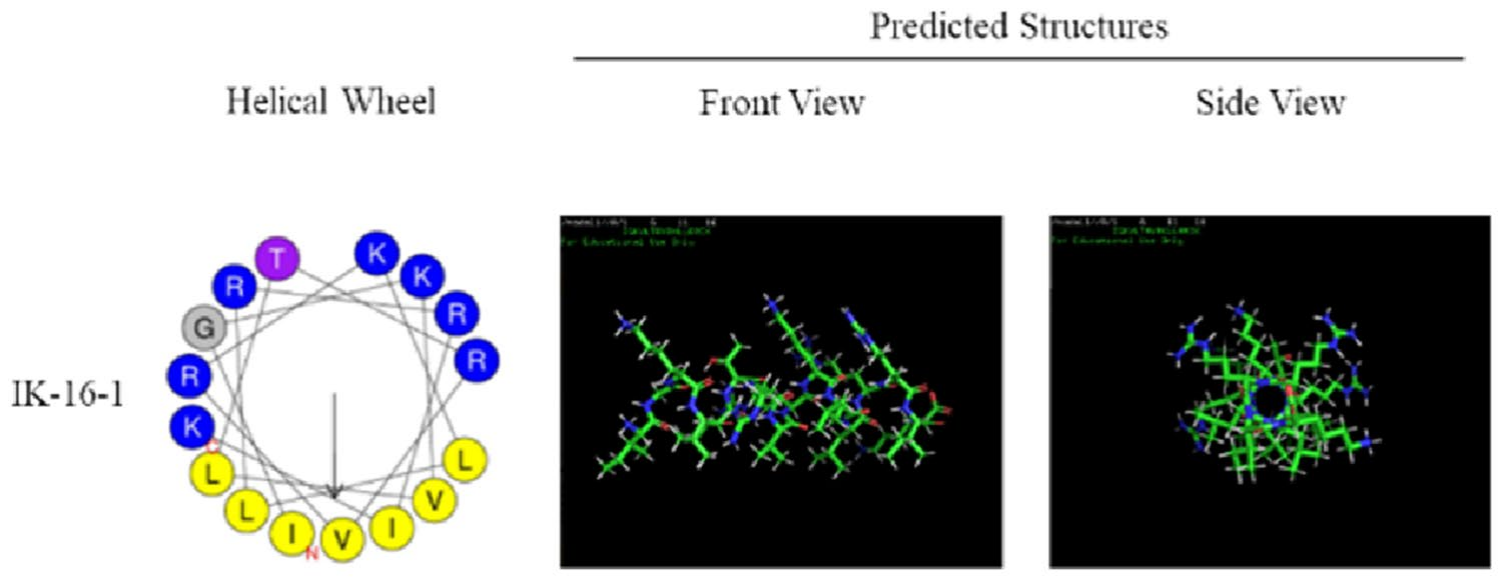
Results of Helical Wheel Projection and predicted structures of beta-defensin analogs.

The AMP was selected from the mesenchymal stem cell secretome and used as a template. Figure 1 illustrates the design process of the antibacterial peptides. We selected the amino acid sequences from the 30th to 45th position of beta-defensin 103 as the template and optimised it to determine the amino acid sequence of the target AMP IGKVLTRVKLLRRIK, named IK-16-1. As depicted in Fig. 2, the predicted structure was an α-helix. The probability score for AMPs was 1, indicating that IK-16-1 is highly likely to become an AMP. The probability of haemolysis was 0.006, indicating that IK-16-1 was less likely to be haemolytic.

### The activities of peptides

To test the inhibitory activity of the peptides, a disk diffusion test and the minimum inhibitory concentration assay was performed. As shown in Table 1, The results showed that IK-16-1 influenced the growth of the four types of microbes. The minimum inhibitory concentration was determined to further test the effects of IK-16-1. As shown in Table 2, IK-16-1 exhibited a broad-spectrum inhibitory effect, although its antifungal activity was not as high as that of antibacterial. Based on the zone of inhibition and minimum inhibitory concentration values, IK-16-1 was found to have broad-spectrum antimicrobial properties against *Escherichia coli*, *Staphylococcus aureus*, *Pseudomonas aeruginosa*, and *Candida albicans*, which are common microbes that cause cosmetic spoilage.

**Table 1:** Zone of inhibition of antimicrobial peptides (AMPs) against different species.

Species	Samples	Diameter (mm)			Mean ± SD (mm)
*Staphylococcus aureus* (18 h, 30 μg)	Kanamycin	26.4	25.5	26.1	26.0 ± 0.4
	IK-16-1	12.3	11.2	12.0	11.8 ± 0.5
*Escherichia coli *(18 h, 30 μg)	Kanamycin	14.6	15.6	15.0	15.1 ± 0.4
	IK-16-1	11.2	11.3	11.5	11.3 ± 0.1
*Pseudomonas aeruginosa *(18 h, 30 μg)	Kanamycin	16.3	15.1	16.0	15.8 ± 0.5
	IK-16-1	11.5	10.7	10.6	10.9 ± 0.4
*Candida albicans *(24 h)	Amphotericin (30 μg)	15.0	15.4	15.2	15.2 ± 0.2
	IK-16-1 (60 μg)	9.6	10.3	8.7	9.5 ± 0.7

*SD* standard deviation.

**Table 2:** Minimum inhibitory concentration (MIC) of IK-16-1 against different species.

Species	MIC (μg/mL, 18 h)			Mean ± SD (μg/mL, 18 h)
*Candida albicans*	125.0	125.0	125.0	125.0 ± 0.0
*Escherichia coli*	125.0	125.0	62.5	104.2 ± 36.1
*Pseudomonas aeruginosa*	125.0	62.5	62.5	83.3 ± 36.1
*Staphylococcus aureus*	125.0	62.5	62.5	83.3 ± 36.1

### The safety of peptides

**Figure 3:**
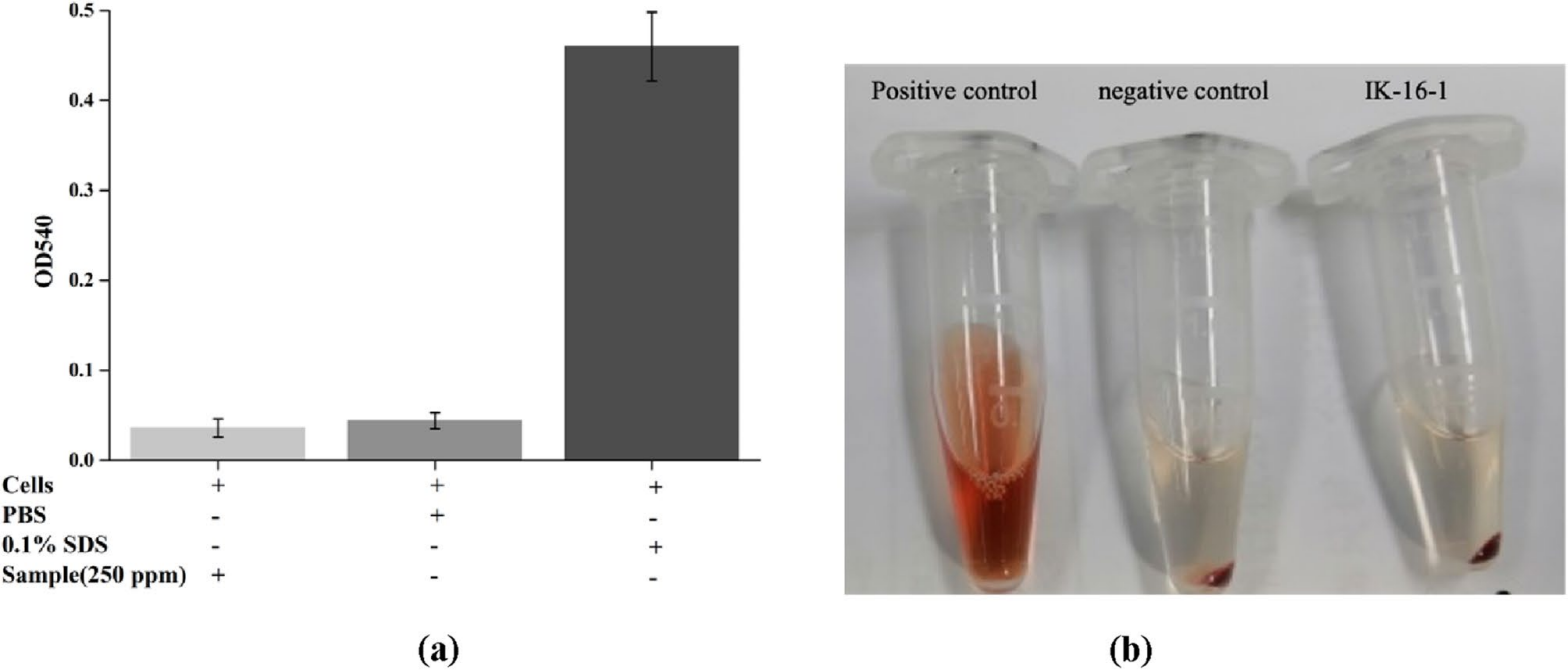
The hemolytic results of IK-16-1 at a concentration of 250 μg/mL (positive control: 0.1% SDS, negative control: PBS).

The cytotoxicity and haemolytic activity of IK-16-1 were evaluated to determine its safety. Compared to the blank group, 10 ppm IK-16-1, a common concentration used in cell models, was found to be non-cytotoxic to keratinocytes. As depicted in Fig. 3, no haemolytic activity was observed even at the highest concentration of the peptide solution used.

### The application of peptides in cosmetic products

To validate the use of IK-16-1 as a synergistic preservative in cosmetics, a common preservative formulation was used in combination with IK-16-1. As shown in Table 3, IK-16-1 significantly inhibited microbial growth at an early stage.

**Table 3:** The colony forming unit (CFU) calculation in cosmetic products at different time.

Species	Day 0 (CFU/mL)	Day3 (CFU/mL)	Day 7 (CFU/mL)	Day 14 (CFU/mL)	Day 22 (CFU/mL)
*Escherichia coli*	< 10	< 10	< 10	< 10	< 10
*Staphylococcus aureus*	< 10	< 10	< 10	< 10	< 10
*Pseudomonas aeruginosa*	< 10	< 10	< 10	< 10	< 10
*Candida albicans*	1.95 × 10	< 10	< 10	< 10	< 10

## Discussion

In the present study, we obtained a novel antibacterial peptide IK-16-1 designed based on β-defensin 103 with significant antibacterial activity and maintaining safety by using machine learning. These results confirmed our hypothesis and verified that the workflow for designing AMPs is feasible. Based on the zone of inhibition and minimum inhibitory concentration values, IK-16-1 was found to have broad-spectrum antimicrobial properties against *Escherichia coli*, *Staphylococcus aureus*, *Pseudomonas aeruginosa*, and *Candida albicans*, which are common microbes that cause cosmetic spoilage. IK-16-1 has a higher antibacterial activity than that of antifungal. IK-16-1 shows good safety, has no cytotoxic to keratinocytes and no haemolytic activity. Notably, the number of preservatives added to cosmetics in the market may exceed the actual requirements. As shown in Table 3, only 30% of the preservatives were used in this study; however, the anticorrosion effect was comparable to that of the normal formulation. The results showed that IK-16-1 effectively killed the microbes in cosmetic products at low concentrations. Therefore, IK-16-1 can be used as a synergistic preservative to reduce the use of preservatives in cosmetics.

AMPs are characterised by both hydrophobic and hydrophilic domains. Most are cationic, and their positive net charge allows them to interact with negatively charged bacterial membranes^44^. Gram-negative and Gram-positive bacteria have molecules on their outer membranes that confer a negative net charge, allowing electrostatic interactions with cationic peptides^45^. AMPs can cause bacterial cell death through both membranolytic and non-membranolytic mechanisms by interacting with intracellular targets such as DNA, RNA, and proteins^19,46–52^. Most AMPs have broad-spectrum antimicrobial activity and are less prone to trigger resistance, which has a good prospect. In this study, peptides with helical structures were adjusted to separate hydrophobic and hydrophilic residues on the surface. Theoretically, the ability of AMPs to form pores could be improved.

ML uses mathematical, statistical, and computational processes to learn from data^53^. Developing new AMPs through ML is an emerging topic. Lee et al. developed a support vector machine (SVM) based classifier to investigate ⍺-helical AMPs and the interrelated nature of their functional commonality and sequence homology^54^. Capecchi et al. trained recurrent neural networks (RNN) using data from the Database of Antimicrobial Activity and Structure of Peptides to design short non-haemolytic AMPs^55^. A recent study used in silico methods to investigate potential AMPs against predatory myxobacteria^56^. The sources of AMPs are diverse. In this study, we designed AMPs based on endogenous human proteins. Leon et al. used computer-aided tools to identify multifunctional peptides with antimicrobial, antibiofilm, and antioxidant potential from plant peptides^57^. Tachapuripunya et al. used biochemical, mass spectrometric, and bioinformatics approaches to comprehensively identify putative bioactive peptides from the mucus proteomes of gastropods^58^. Besides,Ng et al. reported biosurfactants also show antimicrobial properties^59^, which can be considered for cosmetic applications.

Due to the lack of fundamental knowledge of antibacterial mechanisms, the characteristics generated by different ML programs cannot fully characterise the properties of AMPs. However, with the rapid development of screening methods^60^ and the continuous updating of calculation algorithms^61^, challenges in discovering, classifying, and designing AMPs will gradually be solved to provide more alternatives for preventing accidental overgrowth and drug resistance of microorganisms. Notably, wet experiments are the only way to determine the antimicrobial activity of peptides. Integrating ML with targeted experimentation can guide both AMP discovery and design and provide a new understanding of the properties and mechanisms underlying their modes of action^62^.

This study has some limitations. The exact mechanism by which IK-16-1 combines with preservatives to inhibit bacterial growth was not elucidated, and the mechanism through which IK-16-1 interacts with bacteria and other compounds in emulsions remains unclear. Furthermore, whether IK-16-1 has a synergistic effect with other preservatives, such as phenoxyethanol, requires further investigation. Because the performance of IK-16-1 was tested only under emulsion conditions to form its theoretical helical structure, the effects of other dosage forms should be considered in future applications. In future research, we intend to investigate the synergistic effects of IK-16-1 in relation to other preservatives.

Notwithstanding these limitations, this study verified the feasibility of combining computational design with artificial intelligence prediction to design AMPs derived from human endogenous proteins. IK-16-1 inhibits the growth of common spoilage-causing bacteria. Therefore, it can be used synergistically to effectively preserve cosmetics while containing lower concentrations of allergenic preservatives.

## Conclusion

Preservatives, essential ingredients in cosmetic products, are one of the most common allergens. The irritation caused by preservatives has underscore an urgent need for the development of safe and effective broad-spectrum antimicrobial agents. Using machine learning-assisted rational design, we obtained a novel antibacterial peptide IK-16-1 with significant antibacterial activity and maintaining safety based on β-defensins, which could be a promising preservative synergist to reduce the use of allergenic preservatives in cosmetics. This study demonstrated that ML can significantly improve antibacterial activity and safety, shorten development cycles, and reduce development costs.

## Data Availability

All data generated or analyzed during this study are available from the corresponding author (Junxiang Li: lijunxiang@acrdc.cn) on reasonable request.
